# Sickness Absence and Disability Pension in the Very Long Term: A Finnish Register-Based Study With 20 Years Follow-Up

**DOI:** 10.3389/fpubh.2021.556648

**Published:** 2021-03-01

**Authors:** Julia Klein, Kaarina Reini, Jan Saarela

**Affiliations:** Demography Unit, Åbo Akademi University, Vaasa, Finland

**Keywords:** Finland, event history analysis, register data, disability pension, sickness allowance

## Abstract

Sickness allowance is paid for short-term sickness absence and is thus an indicator of temporary ill health, but it is also associated with a heightened risk of receiving disability pension. Using event history analysis, we examined the long-term risk for disability pension receipt after first observed receipt of medically certified sickness allowance in each single year after sickness allowance was first recorded. Utilizing longitudinal data from the Finnish population register, covering the period 1989–2010, we observed 110,675 individuals aged 16–40 years at baseline. Using discrete-time hazard models, we estimated how the first observed receipt of sickness allowance was related to the risk of receiving disability pension, with an average follow-up time of 20.6 years. In this population, about 40 percent received sickness allowance and 10 percent received disability pension. In the first years after sickness allowance receipt, there was a substantial difference between long-term and short-term sickness allowance recipients in the hazard of becoming a disability pensioner. This difference levelled out over time, but even 20 years after the first observed sickness allowance receipt, the hazard of disability retirement was more than 15 times higher than that of non-recipients of sickness allowance. Patterns were similar for men and women. First observed receipt of sickness allowance is a powerful predictor for disability pension receipt, also in the very distant future. Thus, it can be used to monitor people with heightened risk of becoming more permanently ill and falling outside the labour market.

## Introduction

Like in the other Nordic countries, sickness allowance (SA) and disability pension (DP) are important pillars of the social security system in Finland, as they aim to maintain the welfare of people whose health is impaired. A key principle is that a well-functioning system finds a balance between the prevention of income loss due to work-inability and the need to match benefit receipt to actual work disability, while lenient rules may lead to misuse of DP as an early retirement scheme (1). The Finnish system is rather strict, though, as decisions on DP applications are centralised. About 20 percent of all applications are rejected (2), and the relative share of rejections tends to increase (3). DP is nevertheless a major cost factor to society, as return-to-work rates of DP recipients are low (4), with no apparent differences between full DP and partial DP in this respect (5, 6). About half of the Finnish population of 5.5 million persons are aged 25–64 (7). Even though the prevalence of DP has been declining from about 10 percent since the 1990s, currently as many as almost 7 percent of all men and women receive DP (8). The most common causes for being granted DP are mental and behavioural disorders, which account for about half, and musculoskeletal diseases, which account for one fifth of all diagnoses, respectively (9).

Whereas, DP receipt in Finland is not conditional on the receipt of SA, it is usually preceded by some period of receiving SA (10). The risk of becoming a disability pensioner is especially high in the first year after SA receipt (11), and it increases with the length of the sickness spell (12, 13). The most common causes for being granted SA are mental and behavioural disorders, which account for about one third of all sickness days, and musculoskeletal diseases, which account for slightly more than one fourth of all sickness days (14). While different diagnoses of SA are associated with different risks for subsequent DP receipt (15), any period of SA receipt increases the risk for receiving DP in the short term (16). Research on long-term SA recipients in Sweden suggests that the risk of becoming a disability pensioner decreases somewhat as time since SA receipt increases (13).

There is ample evidence about how socioeconomic and demographic characteristics affect SA and DP receipt. The risk for both SA and DP receipt increases with age (17), and so does the risk for DP receipt following SA receipt (18, 19). The risk for DP receipt is negatively related to socioeconomic status (16), although this association weakens with age (12). Marriage and higher income have protective effects (20), and so has employment (13). There are considerable differences between occupational classes in the underlying conditions for SA receipt (15, 21), and in how they translate into the DP risk (11, 16). Women have an overall higher prevalence of sickness absence than men, and they differ in the frequency of diagnoses that lead to SA (22, 23), in the subsequent DP risk (24, 25), as well as in their response to the replacement rate of the SA (26). When examining the association of SA and DP receipt in the long run, the use of longitudinal information on socioeconomic and demographic variables is of vital importance as they are likely to change over time.

In the Nordic context, the association between SA and DP receipt is fairly well-researched, but most studies have had a follow-up time of < 10 years and they have generally analysed the time under risk for DP as one single interval. Therefore, the aim with this study is 2-fold. Firstly, we quantify the association between first observed SA receipt and the DP receipt in the very long term, with a population-based sample in which individuals can be observed for up to 22 years. Thus, these longitudinal register data allow us to follow individuals over a half a working life. Secondly, we analyse the association between SA and DP on a year-to-year basis in order to determine whether and how the risk for DP changes with time since first observed SA receipt. We additionally look at the length of the period of the first observed SA receipt, to analyse whether any difference in DP receipt between short- and long-term SA recipients remains in the very long term. This approach will enable early detection of high-risk groups for DP and, thus, people with elevated risks of permanent poor health.

## Materials and Methods

### Data

The data are based on Statistics Finland's longitudinal employment statistics files (used with permission number TK-53-768-12, all ethical testing has been conducted by Statistics Finland). They contain annual records for 1989–2010, representing individuals residing in Finland in any of these years, and allow for individual follow-up during this period. The dataset consists of a 5 percent random sample of the Finnish-speaking population, plus a similarly constructed 20 percent random sample of the Swedish-speaking population. Both groups are native and have equal constitutional rights. Normalised weights are used to account for the different sampling proportions.

### Sickness Allowance and Disability Pension

In Finland, SA is paid by the Social Insurance Institution of Finland after a waiting period of 10 consecutive working days. It requires a medical certificate of work incapacity from a physician. The amount of SA is based on the income 2 years prior to the sickness spell. The replacement rate is 70 percent with no upper limit. SA is paid for a maximum of 300 working days per same diagnosis over a period of 2 calendar years. If work incapacity remains, DP may be applied for (27). We have approximated the total length of sickness absence per year via annual information on income and amount of SA received. As a sickness period can stretch over the turn of a calendar year, SA receipt in 2 consecutive calendar years was summed up to obtain total length of the SA receipt. To focus on time since first exposure and its long-term associations with DP receipt, we focus on the first SA receipt observed in the data. Time since first observed SA receipt, as measured in years, is consequently the exposure variable in focus.

When a physical or mental condition restricts the working capacity for a person aged under 65, he or she may be eligible for DP. The benefit is granted for a fixed period or indefinitely until age 65 (28). DP is usually preceded by a sickness spell, but it may be granted without a preceding SA period. We have obtained information about DP receipt from records on each person's labour market status as observed at the end of each calendar year.

### Data Restrictions

We have restricted the data to persons who were 16–40 years in 1989, or turned 16 years in 1990 or 1991, which is the lowest age at which SA and DP can be claimed. The follow-up ends at age 60, because many persons start to drop out of the labour market via old-age or other retirement schemes by then (29), at the end of 2010, at death, or at emigration, whichever occurs first (Supplementary Figure 1). These data include 56,272 men and 54,403 women born 1949–1975. The duration of SA receipt is mostly short, that is, at most 2 months (Supplementary Table 1). Among men, 39.2 percent received SA and 9.9 percent received DP (Table 1). Among women, the respective shares are 39.8 percent and 9.1 percent. Among those who received DP, 9.7 percent of men and 9.9 percent of women did so without prior SA receipt (not shown). Among the DP recipients with prior SA receipt, ~10 percent became disability pensioners in the same calendar year they first received SA, and about half received DP within 3 years since first SA receipt (not shown). The mean follow-up time for individuals in the data was 20.6 years.

**Table 1:** Number of individuals, person years, and sickness allowance and disability pension recipients by sex in the data.

	**Men**	**Women**
Number of individuals	56,272	54,403
Number of person years	1,150,056	1,128,826
Number of sickness allowance recipients	22,086	21,667
Number of disability pension recipients	5,557	4,967

### Covariates

The covariates used are standard, known to be related to SA and DP receipt, and have thus been used in previous studies of this kind. They are time varying and refer to the situation at the end of a calendar year (Supplementary Table 1). Age was categorised into nine groups, ages 16–19 and 5-year groups spanning ages 20–24 to 55–59. Family situation distinguished between people living with a partner, those alone, and others. Region of residence separated 20 regions according to the NUTS3 classification, with Helsinki separated from the rest of the surrounding Uusimaa region. Population density of the municipality of residence, which has been found associated with SA and DP rates (16), is according to Statistics Finland's definition, and separated rural, semi-urban, and urban areas. Region of birth, which also correlates with health (30, 31), distinguished Southern, Western, Eastern, and Northern Finland, and persons born abroad. Education was categorised as basic, secondary, and tertiary. Job characteristics have been found to modify the association of SA and DP (16). We classified industry of work into eight categories, and additionally separated persons outside the labour force and unemployed individuals. Income refers to taxable income in quintiles. Homeownership, which is an important socioeconomic indicator in Finland (32), separated persons who lived in self-owned accommodation from others. Language group distinguished between Finnish speakers and Swedish speakers. Both are native and have equal constitutional rights, yet there are marked differences in health outcomes. Swedish speakers have lower age-specific mortality rates (33, 34), and lower rates of SA and DP receipt (17, 30). Calendar year of observation was used to control for any period-specific variation in DP receipt.

### Statistical Analyses

The risk for DP receipt since time of first SA receipt was studied with discrete-time hazard models; see (35) for a similar analytic strategy. The results are presented as hazard ratios with 95 percent confidence intervals. Observations with no recorded SA receipt constituted the reference category for the exposure variable, that is, a person stayed in the reference group until observed receiving SA. The unit of follow-up time was calendar years. Zero on the time-axis referred to the same calendar year. We present unadjusted estimates and estimates adjusted for effects of all the control variables. Separate analyses were undertaken for men and women, as they generally differ in their association of SA and DP receipt (12, 16, 18). The statistical software SPSS 23 was used to conduct the analyses.

As robustness checks, we compared people who entered the observation window at different ages, with different labour market experience, as well as different birth cohorts, and found the results reported here to be largely stable across these alternative specifications.

## Results

**Figure 1:**
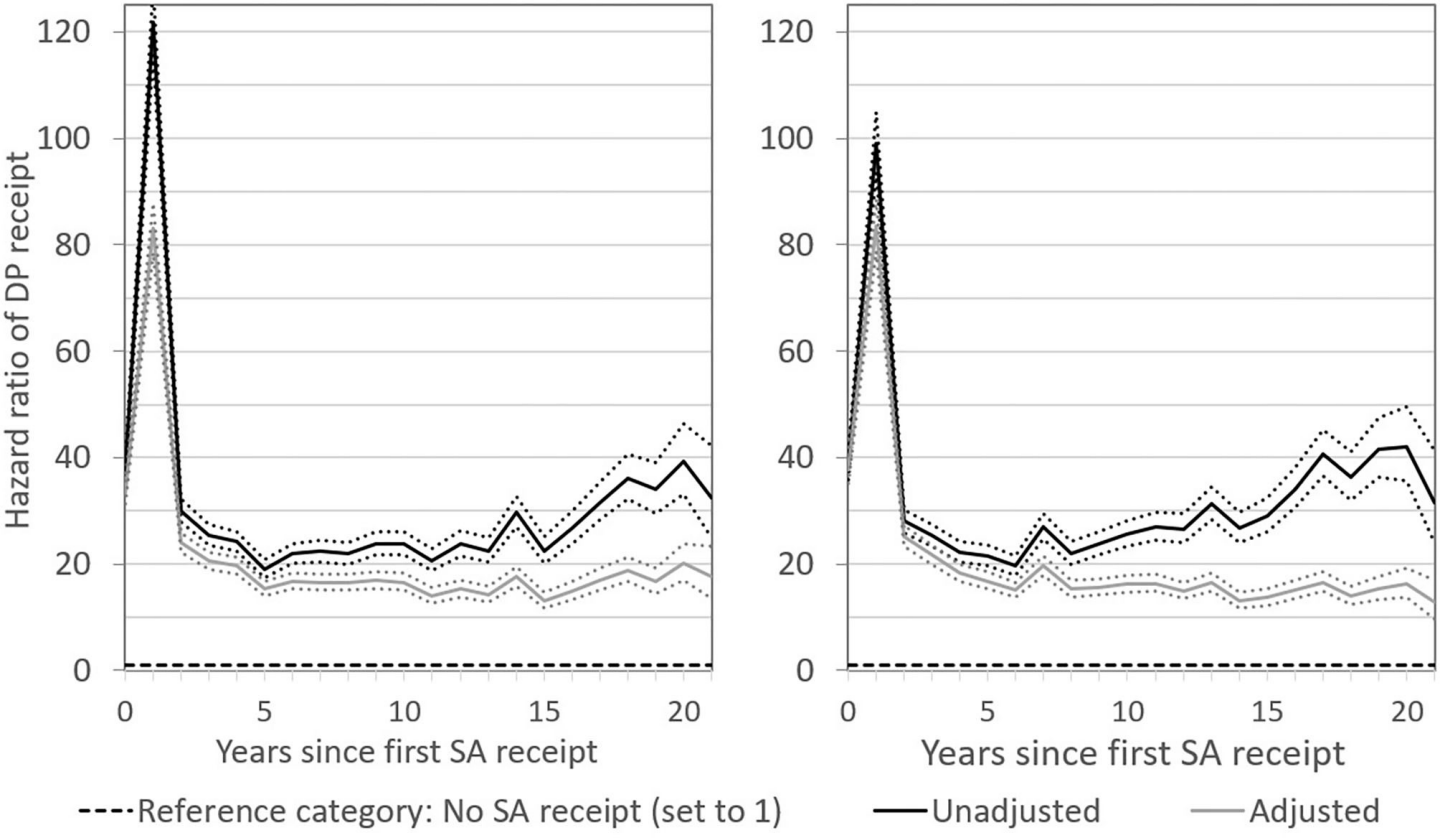
Hazard ratios of DP receipt after first SA receipt in the short and long term. Results for men are presented in the left-hand panel and for women in the right-hand panel. Numbers reported are unadjusted and adjusted estimates with 95 percent confidence intervals.

One year after first SA receipt, the hazard of DP receipt was characterised by a notable peak (Figure 1). This is a reflection of the Finnish health-benefit system in which people move to DP after the exhaustion of SA at 300 days. In the same year as the receipt of SA (year 0), the hazard of DP receipt among men was ~35 times that of no SA being received, according to the adjusted estimates. It increased to a ratio of ~75 times 1 year after and dropped back to about ~25 times 2 years after. After that, SA receipt remained associated with a considerably elevated DP risk also in the very long term. The unadjusted hazard was 20–25 times that of the situation when not having received SA, and it even increased over time since first SA receipt. Adjustment for all the socioeconomic and demographic control variables lowered the hazard ratio somewhat, but even 20 years after first SA receipt, the hazard of becoming a disability pensioner was ~15 times higher as compared to when no SA was received. Short- and long-terms patterns for women appeared to be highly similar to those for men. We performed a likelihood ratio test to see if an adjusted model with interaction between sex and time since SA receipt improved the model fit compared to an adjusted model with main effects of sex and time since SA receipt. The interaction was found to be statistically not significant (*p* = 0.475). With regard to single estimates, the only more marked difference between the sexes was a higher effect size in year 1 in the unadjusted model for men as compared with the corresponding estimate from the unadjusted model for women (Figure 1).

**Figure 2:**
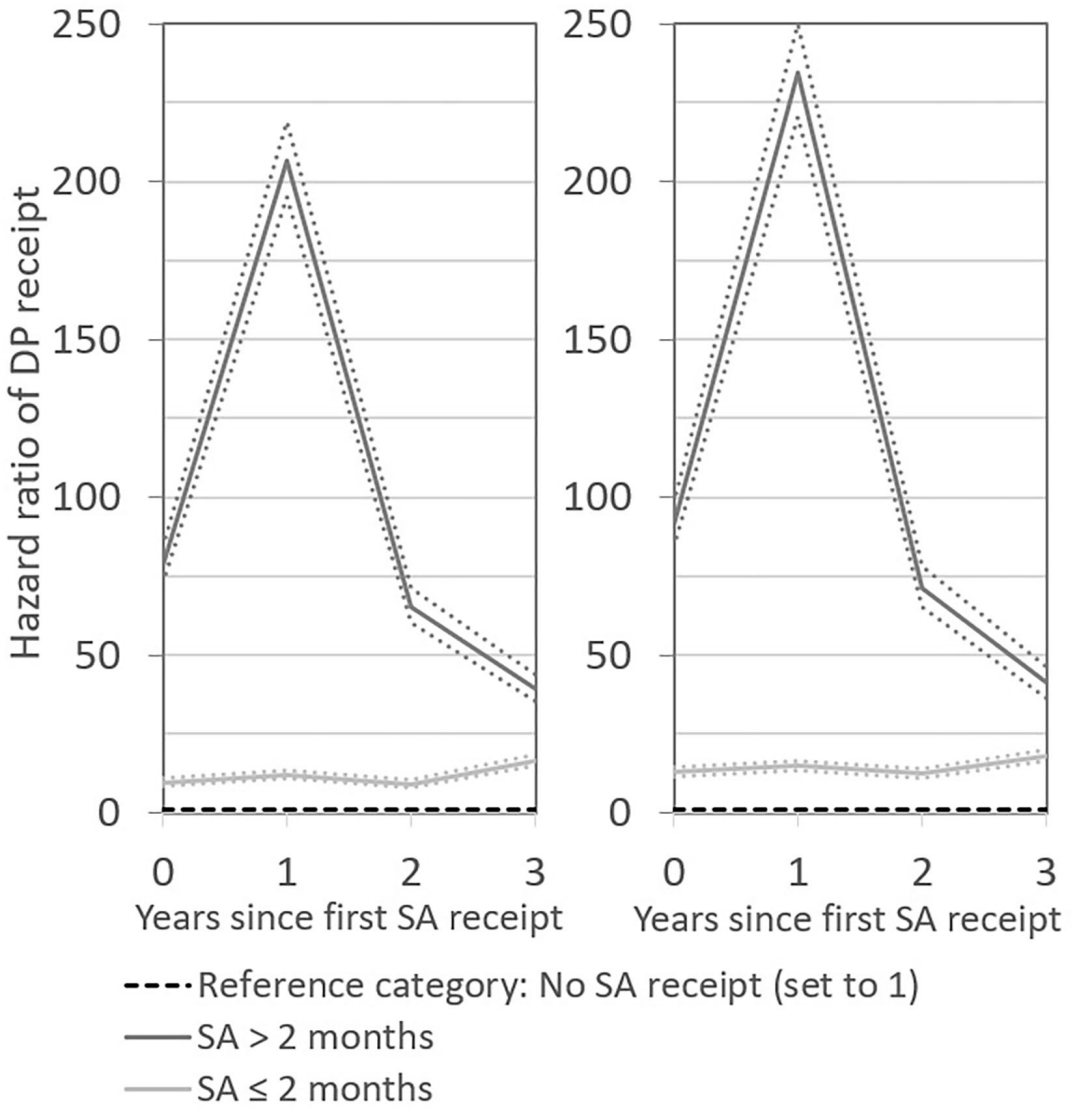
Hazard ratios of DP receipt after first SA receipt by length of SA receipt in the short term. Results for men are presented in the left-hand panel and for women in the right-hand panel. Numbers reported are unadjusted and adjusted estimates with 95 percent confidence intervals.

**Figure 3:**
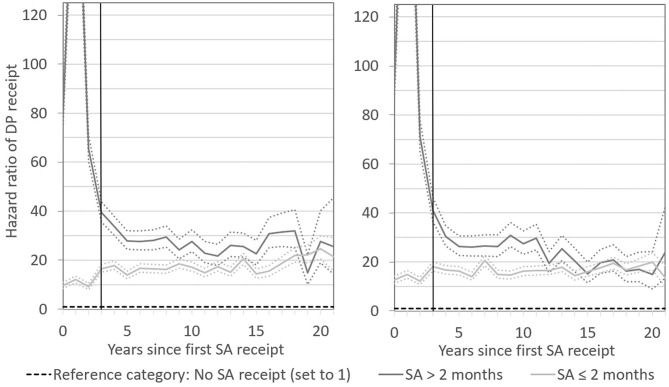
Hazard ratios of DP receipt after first SA receipt by length of SA receipt in the long term. Results for men are presented in the left-hand panel and for women in the right-hand panel. Numbers reported are unadjusted and adjusted estimates with 95 percent confidence intervals. Numbers reported are adjusted estimates with 95 percent confidence intervals. The vertical line in each panel indicates the shift between short- and long-term follow-up at year 3.

When distinguishing the risk for DP receipt by length of SA receipt, and altering the scale of the graph, it became apparent that, for both men and women, the peak in the DP hazard in the first year after SA receipt was primarily related to SA receipt that exceeded 2 months (Figures 2, 3). This was expected, since SA generally is exhausted before DP is applied for. What is more remarkable, however, was the long-term pattern related to short SA receipt. While the DP hazard of men who received up to 2 months of SA was initially much lower than that of men with a longer first SA period, even a short period of SA receipt was associated with a permanently elevated risk for DP, with a hazard ratio of ~15. After ~10 years, the DP patterns for short-term and long-term SA recipients converged. The picture among women was similar. Short SA receipt was not associated with a short-term peak in the risk of DP, but with a steady long-term elevated DP risk, with a hazard ratio of around 15 as compared to non-receipt of SA. In the first 10 years after SA receipt, women who received SA for more than 2 months had a higher DP risk than women who received SA for a shorter period. Thereafter, the risks converged. These patterns suggest that also temporary sickness spells of short-time nature are highly useful for predicting permanent poor health.

## Discussion

We have investigated the association of first observed SA receipt with the risk for DP in the very long term, that is, with a follow-up of more than two decades, and we did so on a year-by-year basis. The very long follow-up is highly relevant from a public health perspective, as it essentially means that we have covered half a working life. The risk for DP was found to be highly elevated in the first year after SA receipt, but even after that, the risk remained considerably high and on a stable level. The short-term peak in the DP risk was driven by long SA periods. In the first 10 years, these persons had a higher DP risk than those with shorter SA periods, but thereafter the DP risk of the two groups largely converged. The results were similar for men and women.

In the short-term perspective, our findings corroborate results from Sweden (11) and Norway (12, 18), which suggest that the DP risk of long-term SA recipients is several-fold higher than that of short-term SA recipients within the time span of a few years. The peak in the DP risk for long-term SA recipients 1 year after first SA receipt is a reflection of the Finnish system, which allows SA receipt for a maximum of 300 working days. A more novel finding is the *absence* of a short-term peak in DP receipt for persons with short periods of SA receipt. To some extent, these results reflect findings from recent research from Finland (16), which has shown that the DP risk increases with the length of a SA spell, particularly if it lasts longer than 180 days. However, the follow-up in that study was no longer than 7 years. In this paper, we have shown that patterns in the correlation of SA with DP receipt for long SA spells and short SA spells converge over time, and that after ~10 years after first SA receipt, they lie at roughly the same high levels.

To our knowledge, this paper is the first which has studied in detail how the interrelation of SA with DP receipt changes as time since SA receipt increases. It has been based on high-quality register data with practically no attrition. Receipt of SA and DP was based on objective assessments made by physicians. The data facilitated a population-based study in order to analyse the DP risk since first observed SA receipt with a very long-term perspective. Previous research on the influence of SA on DP receipt has had a short- or medium-term perspective, meaning that the follow-up period has been two (5), three (12), five (11, 18), seven (16), or up to 10 years (10, 13, 15, 36, 37). Few studies have covered a period exceeding 10 years (19, 38–40). Furthermore, it has been common to analyse some specific cohorts of SA or DP recipients (5, 10, 12, 13, 18, 36). Among studies focussing on only one specific diagnosis for SA receipt (24, 41), follow-up exceeding 10 years has been more common (42, 43). Studies with the longest follow-up, which have analysed the association of all-cause SA on DP, cover ~15 years (19, 38, 39), but have either been based on rather small surveys linked to the population register (19, 39), or on records of one large employer (38). Those results may not be representative for the population at large, which should be considered highly essential from a public health perspective. Population-based studies have been less common (11, 15, 16, 37), and even fewer have had a follow-up that exceeds 10 years. Furthermore, previous studies have also bundled up the time under risk for DP receipt.

SA receipt as observed in our data is a reliable indicator for ill health because it is relying on the certification from a physician. However, the set up did not allow for information on health behaviours, such as smoking (44), which often can be obtained from survey data. Surveys, on the other hand, have the general drawback of self-reporting problems that may stem from selectivity due to ability or motivation to participate, or recall bias (45). Our data had no information about the underlying causes for SA and DP, which somewhat limits our conclusions. Our approximation of the length of SA spells via the fixed reimbursement rate and income may also have led to some inaccuracy. Furthermore, we could not separate full-time and part-time SA periods, although this impediment concerns only the last 3 years of the observation period. Part-time SA was introduced in Finland in 2007, and < 5 percent of all SA recipients received part-time SA in 2007–2010 (14). An avenue for future research could, nevertheless, be to study the causes for SA and DP, together with the part-time solutions, as part-time DP more typically follows part-time SA than full-time DP does, particularly if the return to work fails (5).

Our research has shown that also short SA periods may function as a strong indicator for reduced work ability both in the short term and, even more so, in the very long term. These findings reveal that there is strong negative health selection into the group of first-time SA recipients, and that this selection is pronounced and visible over time, that is, after very many years.

Current policy measures in Finland are correctly emphasising early interventions. In 2012, several legislative changes were introduced and together they are referred to as the 30-60-90 rule (46). This means that employers are obligated to notify the occupational health care provider of their employees' prolonged sickness after 30 days in order to claim daily allowance from Social Insurance Institution of Finland within 60 days, and an assessment by a physician certifying the remaining work ability must be delivered to the Social Insurance Institution within 90 days. In Finland, unemployed persons do not have access to occupational health care. In terms of effective rehabilitation of the whole working age population, initiatives that focus on the prevention of prolonged health problems also among the unemployed should, thus, be favoured.

## Conclusion

Long periods of receiving medically certified sickness allowance signal a heightened risk for disability pension in the short term. However, even short first-time spells of receiving sickness allowance work as a strong signal of reduced work ability, also in the very long term. Men and women are highly similar in the DP risk pattern after SA receipt.

## Data Availability Statement

The data analyzed in this study are subject to the following licenses/restrictions: The data were obtained from Statistics Finland's longitudinal employment statistics files (used with permission number TK-53-768-12). The data are available from Statistics Finland, but restrictions apply to the availability of these data, which were used under license for the current study, and so are not publicly available.

## Ethics Statement

Ethical review and approval was not required for the study on human participants in accordance with the local legislation and institutional requirements. Written informed consent for participation was not required for this study in accordance with the national legislation and the institutional requirements.

## Author Contributions

JK drafted the manuscript and contributed to the analysis of the data. KR conducted the statistical analyses, commented on the manuscript, and contributed to the background and discussion section. JS was responsible for the concept of the study, commented on the manuscript, and contributed continuously in the writing process. All authors read and approve of the final manuscript.

## Conflict of Interest

The authors declare that the research was conducted in the absence of any commercial or financial relationships that could be construed as a potential conflict of interest.

## Supplementary Material

Supplementary Figure 1 Observation plan. Grey shaded area is the observation window.

Supplementary Table 1 Variables' distributions by person years and DP rate, by sex.

## Abbreviations

DP: disability pension
SA: sickness allowance.
