# Supplementary figures and images for: Sickness Absence and Disability Pension in the Very Long Term: A Finnish Register-Based Study With 20 Years Follow-Up
Source: Front Public Health. 2021 Mar 1;9:556648. doi: 10.3389/fpubh.2021.556648 (PMC7956975; doi:10.3389/fpubh.2021.556648)

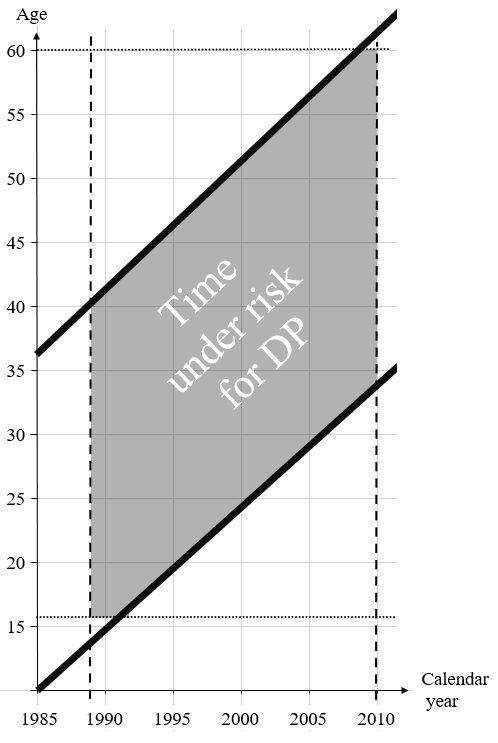

Supplement: Supplementary Figure 1 — Observation plan. Grey shaded area is the observation window. [file Image_1.JPEG]
