# Supplementary material for: Sickness Absence and Disability Pension in the Very Long Term: A Finnish Register-Based Study With 20 Years Follow-Up
Source: Front Public Health. 2021 Mar 1;9:556648. doi: 10.3389/fpubh.2021.556648 (PMC7956975; doi:10.3389/fpubh.2021.556648)
Supplement: Supplementary Table 1 — Variables' distributions by person years and DP rate, by sex. [file Table_1.DOCX]

Table S1: Variables’ distributions by person years and DP rate, by sex

|  | Men | | Women | |
| --- | --- | --- | --- | --- |
| Variables | % | Disability pension rate (%) | % | Disability pension rate (%) |
| Time since SA receipt | | | | |
| no SA | 78.5 | 0.1 | 77.9 | 0.1 |
| year 0 | 2.0 | 2.2 | 2.0 | 2.1 |
| year 1 | 1.9 | 7.4 | 1.9 | 5.5 |
| years 2-4 | 4.7 | 1.7 | 4.8 | 1.5 |
| years 5-9 | 6.2 | 1.4 | 6.4 | 1.4 |
| years 10-14 | 4.2 | 1.6 | 4.4 | 1.6 |
| years 15-21 | 2.5 | 1.9 | 2.6 | 2.0 |
| Length of SA receipt | | | | |
| no SA | 78.5 | 0.1 | 77.9 | 0.1 |
| max 2 months | 17.3 | 1.1 | 18.1 | 1.2 |
| over 2 months | 4.2 | 6.4 | 4.0 | 5.3 |
| Age group | | | | |
| 16-19 | 3.3 | 0.1 | 3.2 | 0.1 |
| 20-24 | 7.7 | 0.1 | 7.6 | 0.1 |
| 25-29 | 11.9 | 0.2 | 11.8 | 0.1 |
| 30-34 | 16.2 | 0.3 | 16.0 | 0.2 |
| 35-39 | 18.3 | 0.3 | 18.4 | 0.2 |
| 40-44 | 17.4 | 0.5 | 17.4 | 0.4 |
| 45-49 | 12.9 | 0.8 | 13.0 | 0.6 |
| 50-54 | 8.4 | 1.4 | 8.6 | 1.2 |
| 55-59 | 3.9 | 2.2 | 4.0 | 2.4 |
| Education | | | | |
| Primary | 24.1 | 0.9 | 19.6 | 0.7 |
| Secondary | 47.7 | 0.5 | 43.1 | 0.5 |
| Tertiary | 28.2 | 0.2 | 37.3 | 0.3 |
| Family situation | | | | |
| With partner | 64.9 | 0.4 | 69.4 | 0.4 |
| Alone | 19.5 | 0.9 | 22.6 | 0.7 |
| Other | 15.6 | 0.4 | 8.0 | 0.3 |
| Region of residence | | | | |
| Helsinki area | 18.2 | 0.4 | 20.4 | 0.4 |
| Rest of Uusimaa | 11.0 | 0.4 | 11.1 | 0.4 |
| Varsinais-Suomi | 8.5 | 0.5 | 8.5 | 0.4 |
| Satakunta | 4.0 | 0.4 | 3.9 | 0.4 |
| Kanta-Häme | 2.9 | 0.5 | 2.8 | 0.5 |
| Pirkanmaa | 7.6 | 0.5 | 7.7 | 0.5 |
| Päijät-Häme | 3.4 | 0.5 | 3.3 | 0.4 |
| Kymenlaakso | 3.3 | 0.6 | 3.2 | 0.5 |
| Etelä-Karjala | 2.3 | 0.7 | 2.2 | 0.5 |
| Etelä-Savo | 2.8 | 0.7 | 2.7 | 0.5 |
| Pohjois-Savo | 4.5 | 0.8 | 4.1 | 0.6 |
| Pohjois-Karjala | 3.0 | 0.5 | 2.7 | 0.5 |
| Keski-Suomi | 4.5 | 0.6 | 4.2 | 0.5 |
| Etelä-Pohjanmaa | 3.2 | 0.6 | 3.2 | 0.5 |
| Pohjanmaa | 6.9 | 0.3 | 6.5 | 0.4 |
| Keski-Pohjanmaa | 1.4 | 0.4 | 1.4 | 0.5 |
| Pohjois-Pohjanmaa | 6.2 | 0.6 | 5.9 | 0.5 |
| Kainuu | 1.6 | 0.7 | 1.5 | 0.5 |
| Lappi | 3.2 | 0.6 | 3.2 | 0.6 |
| Ahvenanmaa | 1.5 | 0.3 | 1.5 | 0.3 |
| Population density | | | | |
| Rural | 25.5 | 0.5 | 23.0 | 0.5 |
| Semi-urban | 33.4 | 0.5 | 33.4 | 0.5 |
| Urban | 41.0 | 0.5 | 43.6 | 0.5 |
| Region of birth | | | | |
| Southern Finland | 30.2 | 0.4 | 30.1 | 0.4 |
| Western Finland | 35.4 | 0.5 | 35.0 | 0.4 |
| Northern Finland | 13.5 | 0.6 | 13.9 | 0.5 |
| Eastern Finland | 19.8 | 0.6 | 20.0 | 0.5 |
| Abroad | 1.1 | 0.3 | 1.0 | 0.2 |
| Job industry | | | | |
| Primary industries | 5.8 | 0.4 | 3.1 | 0.4 |
| Manufacturing | 30.6 | 0.3 | 10.1 | 0.3 |
| Trade, hotel and restaurants | 10.8 | 0.2 | 11.7 | 0.3 |
| Transport and communications | 8.8 | 0.4 | 3.7 | 0.4 |
| Financial and business services | 9.1 | 0.3 | 10.0 | 0.3 |
| Public and other services | 12.9 | 0.3 | 36.7 | 0.4 |
| Outside of labour force | 10.0 | 1.8 | 14.7 | 0.9 |
| Unemployed | 12.0 | 0.5 | 10.0 | 0.4 |
| Income quintiles | | | | |
| 1^st^ | 19.2 | 0.9 | 22.8 | 0.6 |
| 2^nd^ | 15.2 | 0.6 | 26.2 | 0.4 |
| 3^rd^ | 16.9 | 0.4 | 23.5 | 0.4 |
| 4^th^ | 21.8 | 0.4 | 17.0 | 0.4 |
| 5^th^ | 26.7 | 0.2 | 10.5 | 0.3 |
| Homeownership | | | | |
| Homeowner | 72.4 | 0.4 | 70.9 | 0.4 |
| Rented or other | 27.6 | 0.6 | 29.1 | 0.5 |
| Language | | | | |
| Finnish | 82.4 | 0.5 | 82.7 | 0.5 |
| Swedish | 17.6 | 0.3 | 17.3 | 0.4 |
| Observation year | | | | |
| 1989-1992 | 23.6 | 0.2 | 23.4 | 0.1 |
| 1993-1995 | 14.2 | 0.3 | 14.1 | 0.2 |
| 1996-2001 | 27.8 | 0.4 | 27.7 | 0.4 |
| 2002-2005 | 17.8 | 0.7 | 17.9 | 0.7 |
| 2006-2008 | 12.7 | 0.9 | 12.9 | 0.9 |
| 2009-2010 | 3.9 | 0.8 | 4.0 | 0.9 |
